# Distinct Hypoxia-Related Gene Profiling Characterizes Clinicopathological Features and Immune Status of Mismatch Repair-Deficient Colon Cancer

**DOI:** 10.1155/2021/2427427

**Published:** 2021-12-07

**Authors:** Yixin Xu, Junjie Hu, Can Cao, Mili Zhang, Youdong Liu, Haonan Chen, ShanShan Wei, Ziyan Zhu, Yuqin Yang, Liang Yu, Jikun Li

**Affiliations:** ^1^Department of General Surgery, Shanghai General Hospital of Nanjing Medical University, Shanghai, China; ^2^Department of General Surgery, Shanghai General Hospital, Shanghai Jiao Tong University School of Medicine, Shanghai, China; ^3^Department of General Surgery, Wujin Hospital Affiliated with Jiangsu University, The Wujin Clinical College of Xuzhou Medical University, Changzhou, Jiangsu, China; ^4^Department of Gastroenterology, Shanghai General Hospital, Shanghai Jiao Tong University School of Medicine, Shanghai, China; ^5^Department of Laboratory Animal Centre, Shanghai General Hospital, Shanghai Jiao Tong University School of Medicine, Shanghai, China

## Abstract

Despite dramatic responses to immune checkpoint inhibitors (ICIs) in patients with colon cancer (CC) harboring deficient mismatch repair (dMMR), more than half of these patients ultimately progress and experience primary or secondary drug resistance. There is no useful biomarker that is currently validated to accurately predict this resistance or stratify patients who may benefit from ICI-based immunotherapy. As hypoxic and acidic tumor microenvironment would greatly impair tumor-suppressing functions of tumor-infiltrating lymphocytes (TILs), we sought to explore distinct immunological phenotypes by analysis of the intratumoral hypoxia state using a well-established gene signature. Based on the Gene Expression Omnibus (GEO) (*n* = 88) and The Cancer Genome Atlas (TCGA) (*n* = 49) databases of patients with CC, we found that dMMR CC patients could be separated into normoxia subgroup (NS) and hypoxia subgroup (HS) with different levels of expression of hypoxia-related genes (lower in NS group and higher in HS group) using *NMF* package. Tumoral parenchyma in the HS group had a relatively lower level of immune cell infiltration, particularly CD8+ T cells and M1 macrophages than the NS group, and coincided with higher expression of immune checkpoint molecules and C-X-C motif chemokines, which might be associated with ICI resistance and prognosis. Furthermore, three genes, namely, MT1E, MT2A, and MAFF, were identified to be differentially expressed between NS and HS groups in both GEO and TCGA cohorts. Based on these genes, a prognostic model with stable and valuable predicting ability has been built for clinical application. In conclusion, the varying tumor-immune microenvironment (TIME) classified by hypoxia-related genes might be closely associated with different therapeutic responses of ICIs and prognosis of dMMR CC patients.

## 1. Introduction

Colon cancer (CC) is the most prevalent type of malignancy worldwide, resulting in the fifth leading cause of death in 2020 [1]. The standard therapeutic procedures for advanced CC are surgery plus adjuvant therapy or neoadjuvant therapy. However, from 2018 to 2021, the death rate of patients with advanced disease has almost not changed [1, 2]. One of the molecular subtypes of CC, characterized by higher tumor mutational burden (TMB), more neoantigens, and relatively favorable outcomes, is deficient mismatch repair (dMMR). A previous clinical trial demonstrated that metastatic patients with CC with dMMR tumors exhibited impressive and deep pathological responses to ICI-based immunotherapy [3]. However, approximately 60% of dMMR patients may still progress and experience resistance to the same regimens [4]. Thus, many studies have focused on conducting comprehensive genome-wide analysis to identify the underlying molecular mechanisms of poor response to ICIs and distinct outcomes in other types of human malignancy [5–7].

Some studies reported that higher level of TMB and large production of neoantigens were closely associated with the infiltration of immune cells and thus the response rate to immunotherapy [8], whereas many factors, such as restrained oxygen and nutrients, may impact tumor-infiltrating lymphocytes (TILs), leading to diverse and complex TME [9, 10]. One major player significantly influences many types of cells in TME is hypoxia. Although hypoxia is considered a hallmark of TME, it is widely known as a suppressor for immune cell metabolism and a promotor for tumor development and progression [11, 12]. Tumors and those tumor promotors deprive of oxygen and nutrient and subsequently produce hypoxia and acidic TME, which restrain the function of those TILs [13]. For example, the increased expression of lactic acid, which is the typical feature of hypoxia TME, plays an important immunosuppressive role by restraining the metabolism of TILs and thus inhibits the production of interferon-*γ* (IFN-*γ*) [14, 15]. Besides, another enriched expressed factor in hypoxia TME, named hypoxia-inducible factor-1*α* (HIF-1*α*), impairs the proliferation, migration, and cytotoxic function of CD8+ T cells by promoting them to a glycolytic phenotype [16].

In this study, based on the Gene Expression Omnibus (GEO) and The Cancer Genome Atlas (TCGA) databases, we have investigated the role of hypoxia that acted in the tumor-immune microenvironment (TIME) of patients with CC with dMMR. Using hypoxia-related gene signature, we have classified dMMR patients into 2 subgroups and evaluated their different TIMEs. Moreover, we have also developed a prognostic model for dMMR CC patients in clinical application.

## 2. Materials and Methods

### 2.1. Datasets for Molecular Classification and Validation

The microarray data of 88 dMMR CC patients were collected from GSE39084 (16) and GSE39582 (72), which belonged to the Gene Expression Omnibus (GEO) database (https://www.ncbi.nlm.nih.gov/geo/). Because the number of dMMR CC patients was not enough for analysis, we merged both datasets after the batch effect was eliminated using *limma* (version 3.48.1) and *sva* (version 3.40.0) packages. Meanwhile, data of 49 dMMR CC patients for validation were obtained from The Cancer Genome Atlas (TCGA) database (https://portal.gdc.cancer.gov/) using GDC API tools on July 7, 2021.

### 2.2. Hypoxia-Related Gene List

This list contained 200 hypoxia-related genes, which were accessed from the HALLMARK_HYPOXIA gene set of the Gene Set Enrichment Analysis (GSEA) database (http://www.gsea-msigdb.org/gsea/msigdb/cards/HALLMARK_HYPOXIA.html) [17, 18]. The complete gene list was contained in Table S1.

The immune-related genes were obtained from the Tracking Tumor Immunophenotype database (http://biocc.hrbmu.edu.cn/TIP/index.jsp) [19]. This list contained negative, positive, T cell, CD8+ T cell, CD4+ T cell, dendritic cell, eosinophil, macrophage, monocyte, neutrophil, nature kill (NK) cell, Th1 cell, Th17 cell, Th 2 cell, Th22 cell, and Treg cell-correlated genes.

### 2.3. Procedure of Clustering Analysis

First, we used univariate Cox analysis to identify the prognostic value of hypoxia-related genes. Then, genes with *p* < 0.05 were chosen for subsequent analysis. The combined gene set, which contained 88 dMMR CC patients, was clustered using an unsupervised nonnegative matrix factorization (NMF) (version 0.23.0) package [20]. The optimal clustering number was chosen according to the *k* value when the cophenetic correlation coefficient started to decline. Then, the clustering result was evaluated using principal component analysis (PCA) and t-distributed stochastic neighbor embedding (t-SNE). Meanwhile, the overall survival (OS) of different subgroups was evaluated according to the result of the Kaplan–Meier analysis that was performed using the *survival* (3.2–11) package. Moreover, the clustering method according to hypoxia-related genes was validated using the TCGA COAD cohort.

### 2.4. Identification of Differentially Expressed Genes and Functional Annotation Analysis

Differentially expressed genes (DEGs) with FDR < 0.05 and |log_2_FC| > 1 were identified between 2 different subgroups using *limma* (version 3.48.1) package. Moreover, the functions according to DEGs were evaluated through the Gene Ontology (GO) and Kyoto Encyclopedia of Genes and Genomes (KEGG) using *clusterProfiler* (version 4.0.2), *topGO* (version 2.44.0), and *pathview* (version 1.32.0) packages.

### 2.5. Evaluation of Tumor-Immune Microenvironment

To investigate the differences in TIME between the 2 subgroups, we used CIBERSORTx to estimate the infiltration of 22 different immune cells (https://cibersortx.stanford.edu/) [21]. Meanwhile, we also extracted the expressions of immune checkpoint molecules and CXC chemokines from microarray data of GEO sets and RNA-seq data of the TCGA cohort. The expressions of these variates were compared between different subgroups using the Wilcoxon test.

### 2.6. Construction of Prognostic Model Based on Prognostic Hypoxia-Related Genes

After molecular clustering, we built a prognostic model based on 2 hypoxia-related hub genes, which were the intersection of the GEO-DEGs and TCGA-DEGs using the least absolute shrinkage and selection operator (LASSO) regression analysis. The formula of risk score model was built as follows:

(1) $$\begin{array}{c} \text{Risk score model}=\sum_{i}\beta_{i}\;*\text{ hub }{\text{gene}}_{i}. \end{array}$$

The *i* index represents a significantly prognostic gene of the Lasso regression analysis, and *β*_*i*_ stands for the beta coefficients of these genes.

### 2.7. Model Validation and Visualization

The discrimination ability of the prognostic model was assessed using receiver operating characteristic (ROC) curve analysis. It would calculate the true positive (TP) and false positive (FP), based on a series of different binary classification methods (critical or cutoff value). The curve was drawn with the TP or sensitivity as the ordinate, and with FP or 1-specificity as the abscissa. The area under the curve (AUC) was used for quantitative analysis in ROC analysis. Generally, (1) AUC between 0.5 and 0.7 would be considered as lower accuracy; (2) AUC between 0.7 and 0.9 would be considered to be valuable; and (3) AUC above 0.9 would be considered as high accuracy; however, AUC = 0.5 meant that the model had no diagnostic value. In this study, ROC analysis was performed using the *timeROC* package (version 0.4).

Based on the Cox proportional hazard model, the calibration plot was used to assess the stability of the prognostic model. Meanwhile, the Kaplan–Meier (K-M) survival analysis was performed according to different risk scores (low-risk score vs. high-risk score). The calibration and K-M analysis were performed using the *rms* (version 6.1-0) and *survival* packages.

To visualize the prognostic model, we used *rms* (version 6.1-0) to create a nomogram that could estimate the 1-, 3-, and 5-year OS of dMMR CC patients. In addition, to make the prediction model more user-friendly, we developed an online dynamic calculator application using *shiny* (version 1.5.0) and *DynNom* (version 5.0.1) packages in R. This online calculator allows users to input their characteristics, and then, it will automatically export an outcome of the OS (https://xyxdoctor.shinyapps.io/OS_of_dMMR_Colon_Cancer/).

### 2.8. Statistical Analysis

Continuous and categorical (frequencies and percentages) variables were analyzed using independent *t*, chi-square, or 2-tailed Fisher's exact test, respectively. Meanwhile, ranked data were analyzed using the Mann–Whitney *U* test. The discrimination of the prediction model was assessed using ROC analysis. The OS was defined as the period from the date of surgery to the date of death due to any cause. OS between different groups was measured using the log-rank method of K-M analysis. Cox regression analysis was used to assess the time event-dependent OS status of CC dMMR patients. The correlation of RNA expression among different hub genes was measured using Spearman's analysis. A *P*-value less than 0.05 was considered statistically significant. All statistical analyses were carried out using *R* software (version 4.0.3; https://www.r-project.org/) and *R* studio (version 1.3.1093; https://www.rstudio.com/) software.

## 3. Results

### 3.1. Different Characteristics between Different Molecular Subgroups

**Figure 1:**
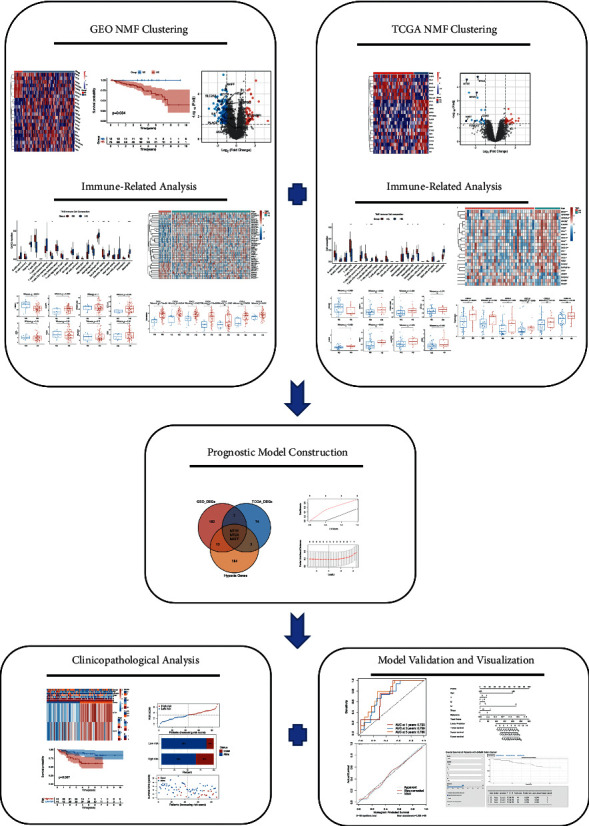
The complete pipeline of this study.

**Figure 2:**
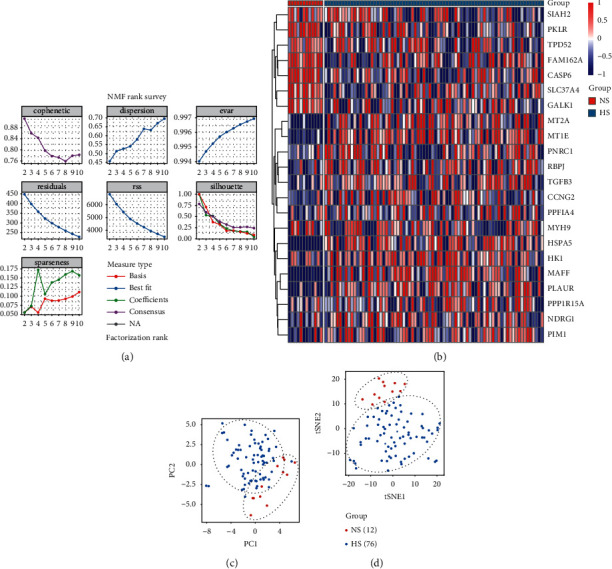
The procedure of identifying two distinct hypoxia-related molecular subtypes for dMMR CC patients. (a) Factorization rank for *k* = 2–10. (b) The heatmap identified that the expression of 22 prognostic hypoxia-related genes was different between the two clusters. The PCA (c) and t-SNE (d) revealed that the clustering method based on the hypoxia-related genes could clearly classify the patients into two groups.

The complete pipeline of this study is shown in Figure 1. Initially, we filtered 22 genes that were associated with the prognosis of dMMR patients from the 200 hypoxia-related gene list obtained from the GSEA database (Table S2). Based on these genes, we classified the GEO set (GSE39084 + GSE39582) using the *NMF* package. The optimal clustering number was chosen according to the *k* value, which was determined by the cophenetic correlation coefficient. In this study, the cophenetic correlation coefficient started to decrease, when the *k* value was 2 (Figure 2(a)). Then, we evaluated the differential expressions of 22 prognostic hypoxia-related genes in different subgroups. The results showed that most of the hypoxia-related genes were increasingly expressed in Cluster2 (Figure 2(b)). Meanwhile, the distributions of patients from different subgroups were separated clearly *via* PCA and t-SNE analyses (Figures 2(c) and 2(d)). Combined with these results, we finally decided to classify 88 dMMR CC patients into 2 different subgroups. Cluster1 was defined as normoxia subgroup (NS), and Cluster2 was defined as hypoxia subgroup (HS).

**Figure 3:**
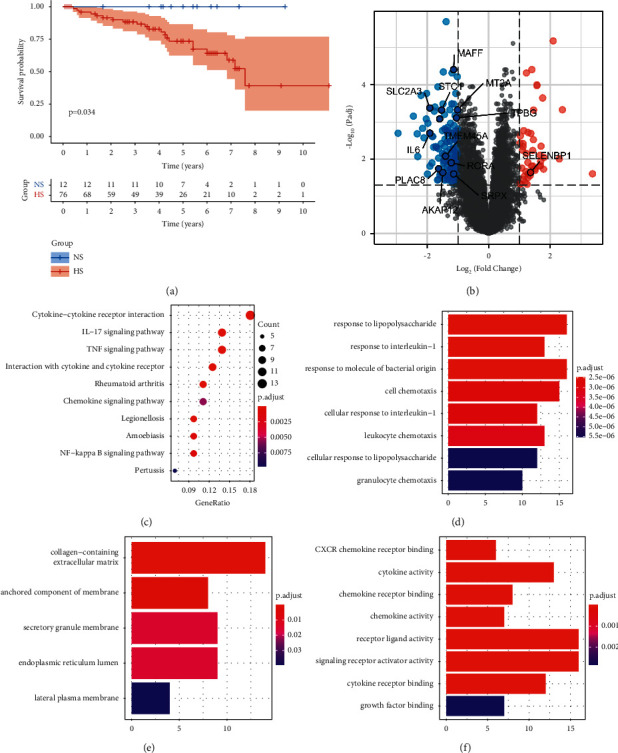
The differences in survival status and functional enrichment between the two subgroups. (a) Kaplan–Meier curve showed that NS had significantly better OS than HS. (b) There were 198 DEGs between two subgroups. Among these DEGs, 13 genes were hypoxia related, which were shown with their names. (c) The KEGG signaling pathways showed that these DEGs enriched in cytokine, TNF, chemokine, and NF-*κ*B pathways. Biological processes (d), cellular component (e), and molecular function (f) also showed similar results with KEGG analysis.

The clinicopathological features between different subgroups were also analyzed (Table S3). We found that patients in NS were younger (*p* < 0.001) and had better pathological stages (*p*=0.038). In the TCGA COAD cohort, the pathological stage and *M* were also better in NS. In the combined GEO cohort, all 88 patients with CC had complete follow-up information. We used K-M analysis to compare their prognosis between NS and HS. The results showed that the OS of NS was significantly better than that of HS (*p*=0.034) (Figure 3(a)). Interestingly, none of the patients in NS had died during follow-up. In coincidence with our findings, previous studies also found that pathological stage and age were closely associated with the OS/DFS of patients with colorectal cancer (CRC) [22, 23].

After the distinct clinical features of the two subgroups were uncovered, we decided to investigate whether differences also existed on gene expression and functional levels. We found that 198 genes were differentially expressed (|log_2_FC| > 1 and FDR < 0.05) between NS and HS (Figure 3(b)). Among them, the expression of 153 genes was significantly lower and the expression of other genes was higher in NS, compared to HS. Meanwhile, 12 hypoxia-related genes, including MAFF, MT1E, MT2A, et al., were decreasingly expressed in NS, and only one hypoxia-related gene, named SELENBP1, was increasingly expressed in NS. This result was consistent with the clustering result shown in Figure 2(b), which indicated that most hypoxia-related genes were decreasingly expressed in NS compared to those in HS. Based on the KEGG analysis, we found that these DEGs were involved in inflammation-related (cytokine-cytokine receptor interaction, chemokine signaling pathway, and NF-*κ*B signaling pathway) and tumor-related (TNF signaling pathway) pathways (Figure 3(c)). Similarly, the result of biological processes (Figure 3(d)), cellular component (Figure 3(e)), and molecular function (Figure 3(f)) analysis using GO showed that these DEGs were associated with inflammatory function, including response to interleukin-1, cytokine activity, chemokine activity, CXCR chemokine receptor binding, and others. Taken together, these results indicated that the hypoxia condition in TME would not only regulate the biological behavior of tumor cells but also change the metabolism and secretion of immune cells. These findings were also supported by previous studies [24–26]. They found that the deprival of oxygen and glycose caused by the tumor and stromal cells would induce exhaustion and dysfunction of TILs, subsequently creating an immunosuppressive TME.

### 3.2. Different Tumor-Immune Microenvironments between Subgroups

**Figure 4:**
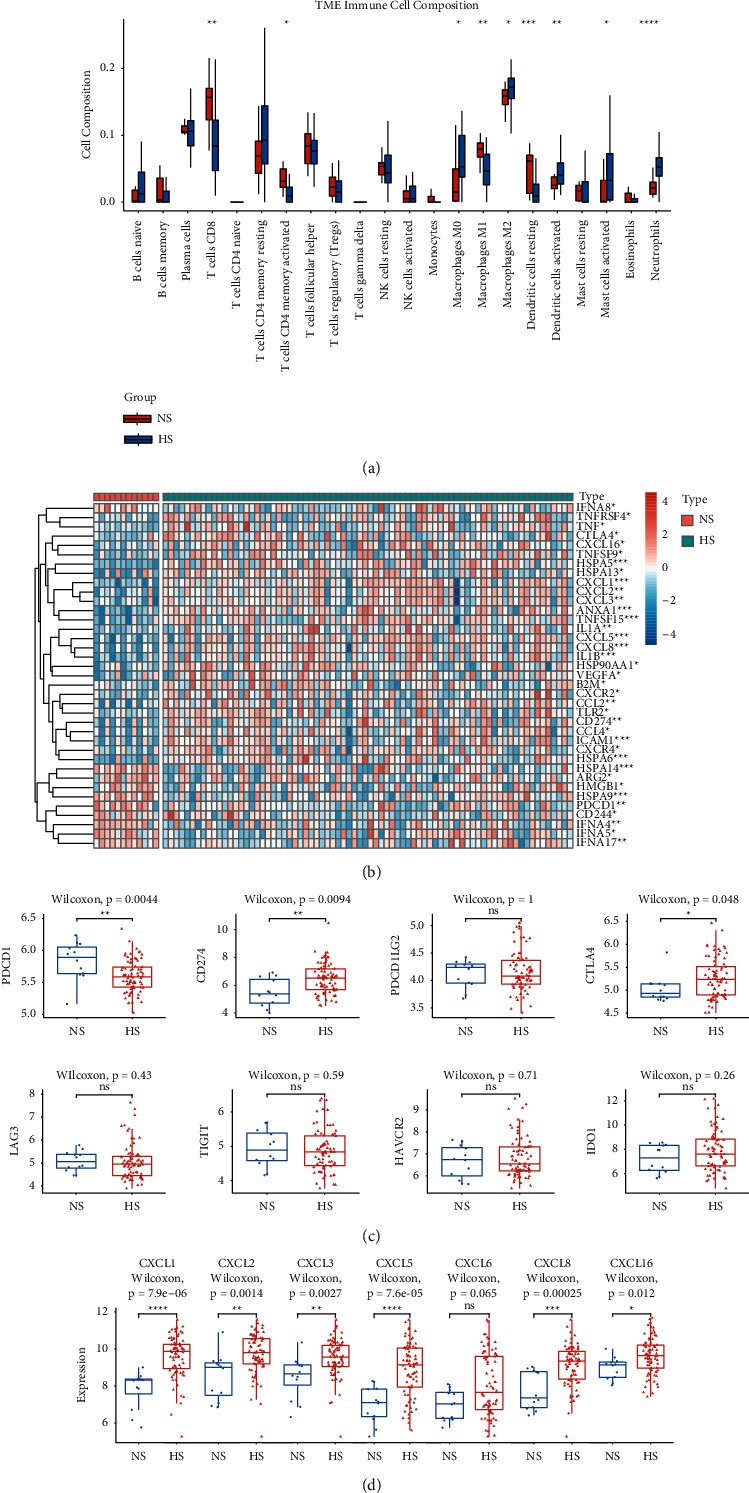
The analyses of TIME identified that two subgroups had a quite different infiltration of immune cells and the expression of immune-related genes, immune checkpoint molecules, and chemokines. (a) The result of CIBERSORTx analysis showed that NS had higher infiltration of CD4+ T cells, CD8+ T cells, and M1 macrophages and lower infiltration of M2 macrophages and neutrophils. (b) The heatmap revealed different expressions of immune-related genes between two subgroups. (c) Despite PDCD1, all the other immune checkpoint molecules were increasingly expressed in HS. (d) Most of the chemokines were also increasingly expressed in HS. ^*∗*^represents *p* < 0.05, ^*∗∗*^represents *p* < 0.01, ^*∗∗∗*^represents *p* < 0.001, and ^*∗∗∗∗*^represents *p* < 0.0001.

Because the survival status was significantly different between the two subgroups and the functions of the DEGs were enriched in inflammatory pathways, we decided to investigate whether the TIME between the two subgroups was distinct. Through analysis of CIBERSORTx, we found that the resting of CD8+ T cells, CD4+ T cells activated, M1 macrophages, and dendritic cells resting were significantly more in NS (Figure 4(a)). We supposed that the relative normoxia TME might be conducive to the infiltration of cytotoxic T cells and the polarization of macrophages into the M1 type. Previous studies suggested that HIF-1, a major factor elevated in hypoxia zone and PD-L1, could directly regulate the differentiation of tumor-associated macrophages (TAMs) and convert them into polarized M2 type, which often played an immunosuppressive role in TME and led to drug resistance of immunotherapy [27–29].

To more deeply investigate the differences of TIME, we also evaluated the expressions of immune-related genes (obtained from the Tracking Tumor Immunophenotype database), immune checkpoint molecules, and C-X-C motif chemokines between two subgroups. The result showed that most interferon (IFN)-related genes were increasingly expressed in NS, which followed the previous result that CD8+ and CD4+ T cells were significantly richer in NS (Figure 4(b)). Despite PDCD1, also known as programmed cell death 1 (PD-1), all the other immune checkpoint molecules were decreasingly expressed in NS (Figure 4(c)). Although PD-1 was expressed by types of immune cells, including B cells, natural killer cells, innate lymphoid cells, and myeloid cells, its expression mainly occurred on T cells [30]. In our study, we found that most types of T cells, including CD8+, CD4+ memory activated, follicular helper, and regulatory T cells, were enriched in NS compared to those in HS. These might be associated with the increased expression of PD-1 in NS. Similar to the result shown in the heatmap of Figure 4(b), the chemokine-related genes, including CXCL1, 2, 3, 5, 8, and 16, were significantly increasingly expressed in HS (Figure 4(d)). Korbecki et al. suggested that the elevated expression of CXC chemokines was associated with hypoxia TME and also related to the poor prognosis of patients [31].

We also used the data of dMMR CC patients from the TCGA COAD cohort to validate the hypoxia-related gene clustering method (Figure S1). The heatmap plot showed that the expressions of prognostic hypoxia-related genes were different in different subgroups. Meanwhile, there were 81 differentially expressed genes between subgroups and 6 DEGs were hypoxia related. Consistent with previous results obtained in the GEO sets, the analyses in the TCGA COAD cohort revealed that except for PD-1, the expressions of most immune checkpoint molecules were lower in NS. Moreover, the CXC chemokines were increasingly expressed in HS, which was also in accordance with the result from GEO sets.

Taken together, most T cells, especially CD8+ and CD4+ memory-activated T cells, in coincidence with the expression of IFN-related genes, were enriched in NS. The elevated expression of PD-1 might be related to a higher level of colonization of T cells, whereas increasing expression of immune checkpoint molecules was closely associated with immunosuppressive TME and resistance of ICIs [32]. In our study, despite PD-1, all the other immune checkpoint molecules were increasingly expressed in HS. Combined with a higher level of CXC chemokines, patients in HS might have a relatively immunosuppressive TME, poorer response to immunotherapy, and worse prognosis compared to those in NS.

### 3.3. Analyses for Hypoxia-Related Hub Genes

**Figure 5:**
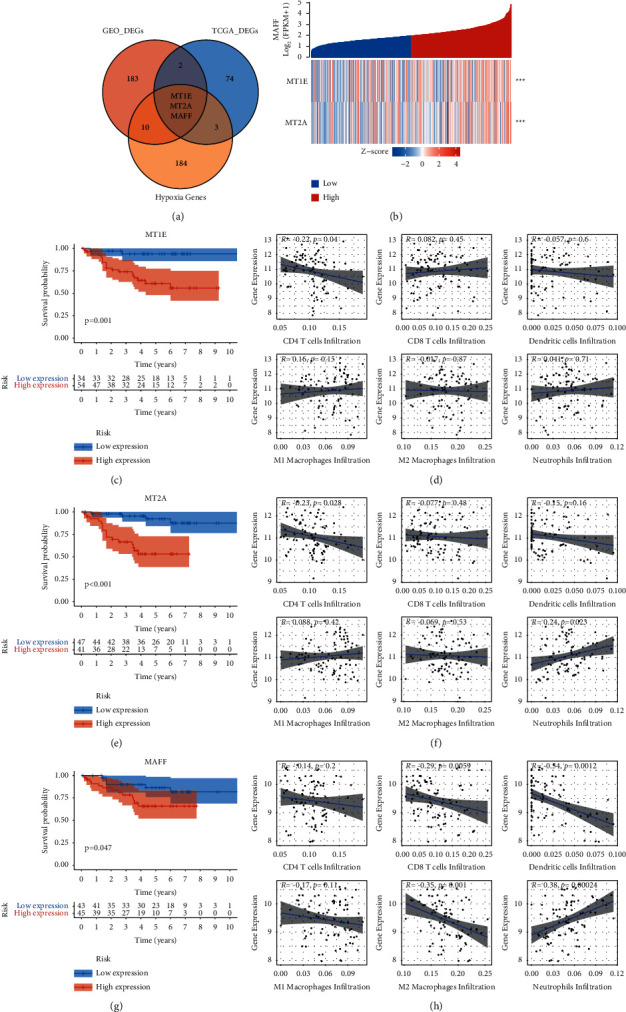
The analyses about three hub genes, such as MT1E, MT2A, and MAFF. (a) Three hub genes were the intersection of the DEGs of GEO and TCGA sets and 200 hypoxia-related genes. (b) The expression of these genes was significantly positively correlated. The Kaplan-Meier curves of MT1E (c), MT2A (e), and MAFF (g) showed that higher expression of these genes was significantly associated with poorer prognosis of dMMR CC patients. The spearman correlation plots of MT1E (d) and MT2A (f) showed similar results: the expression of MT1E and MT2A was negatively related to the infiltration of CD8+ T cells. (h) Meanwhile, the expression of MAFF was negatively associated with the infiltration of CD4+ T cells, CD8+ T cells, dendritic cells, and M2 macrophages, and positively associated with the infiltration of neutrophils. ^*∗*^represents *p* < 0.05, ^*∗∗*^represents *p* < 0.01, ^*∗∗∗*^represents *p* < 0.001, and ^*∗∗∗∗*^represents *p* < 0.0001.

In previous analyses, we found that there were 198 DEGs between NS and HS in GEO sets and 81 DEGs in the TCGA COAD cohort. We wondered whether there were intersections between DEGs in GEO sets and DEGs in the TCGA cohort. The Venn plot showed that 5 genes were differentially expressed in both GEO sets and TCGA cohort (Figure 5(a)). Among them, 3 genes, namely, MT1E, MT2A, and MAFF, were hypoxia related.

Next, we investigated the correlation of the expression of these genes. The expression correlation plot suggested that all three genes were positively expressed (*p* < 0.001) (Figure 5(b)). Since all three genes were increasingly expressed in HS in both GEO and TCGA cohorts, we supposed that these genes would be negatively related to the OS and the infiltration of immune cells. Therefore, we evaluated the correlation using K-M and Spearman's analyses (Figures 5(c)–5(h)). The results showed that increasing expression of three genes was significantly associated with poor OS status (Figures 5(c), 5(e), and 5(g)). Meanwhile, all 3 genes were negatively related to the infiltration of CD4+ *T* and dendritic cells and positively related to the infiltration of neutrophils. Previous studies also identified that higher infiltration of neutrophils was consistent with poor survival status in numerous types of cancer [33–35]. Moreover, the expression of MAFF was negatively associated with the infiltration of CD8+ T cells. Since MAFF was increasingly expressed in HS, which also had fewer infiltrations of CD8+ T cells, CD4+ T cells, and M1 macrophages, and more infiltration of M2 macrophages and neutrophils, it revealed that MAFF might be the potentially important regulatory gene in hypoxia TIME.

### 3.4. Construction of Prognostic Model Based on Hypoxia-Related Genes

The previous analyses identified that dMMR CC patients could be classified into hypoxia and normoxia subgroups. These subgroups showed different expressions of hypoxia-related genes, functional enrichments, TIME, and prognosis. Three genes, namely, MT1E, MT2A, and MAFF, were differentially expressed between NS and HS in both GEO and TCGA cohorts. Next, we wondered whether a prognostic model based on these genes could be built for clinical application.

**Figure 6:**
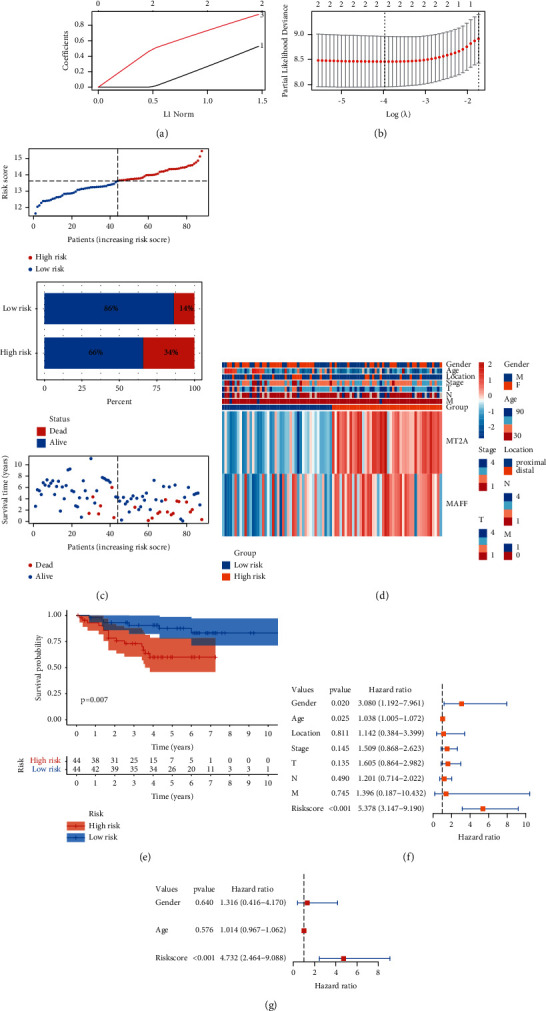
Construction of prognostic model based on 2 prognostic hypoxia-related genes. (a) Twenty-time cross-validation for tuning parameter selection in the LASSO Cox model. The plot of LASSO coefficients (b) showed that the best choice of the number of these genes was 2. (c) The risk score rank (up) and distribution of survival status (middle and down) showed different risk scores and survival status between low- and high-risk groups. (d) The heatmap showed the differences in clinicopathological features and the expression of 2 selected genes between low- and high-risk groups. (e) The Kaplan–Meier curve showed significantly different OS between the two subgroups. The univariate (f) and multivariate (g) Cox analyses showed that the risk score was the independent prognostic factor of dMMR CC patients.

Initially, 3 genes were included. Through Lasso Cox regression analysis, 2 genes, such as MT2A and MAFF, were filtered for the construction of this prognostic model (Figures 6(a) and 6(b)). The formula of the prognostic model was shown as follows:

(2) $$\begin{array}{c} \text{Risk score}=0.861\times \text{MT}2\text{A}+0.426\times \text{MAFF }\left(\text{the median of risk score was }13\text{.}551\right). \end{array}$$

We defined groups with higher scores than the median as a high-risk group, while lower scores than the median as a low-risk group. From the risk plot (Figure 6(c)) and K-M curve (Figure 6(e)), we found that the low-risk group had a lower ratio of dead cases and a significantly better prognosis (*p*=0.007). Meanwhile, the low-risk group had lower expression of hub genes, better pathological *N*, *T*, stage, younger age, and more cases of female patients and distal colon tumors (Figure 6(d)). Through univariate and multivariate Cox analyses, we identified that the prognostic model was the independent predictor for dMMR CC patients (Figures 6(f) and 6(g)).

The clinical characteristics of the low-risk group were distinct from the high-risk group, including the proportion of gender, age, tumor location, and pathological features. Our findings were supported by previous studies [36, 37]. They also found that male patients, right-side tumors, and poor pathological features were potential risk factors for recurrence and prognosis of patients with colorectal cancer (CRC). On the other hand, it also indicated that two genes, namely, MT2A and MAFF, might act an important role in the development and progression of colon cancer and the prognostic model based on these genes would be valuable for clinical application.

### 3.5. Calibration and Visualization of the Prognostic Model

**Figure 7:**
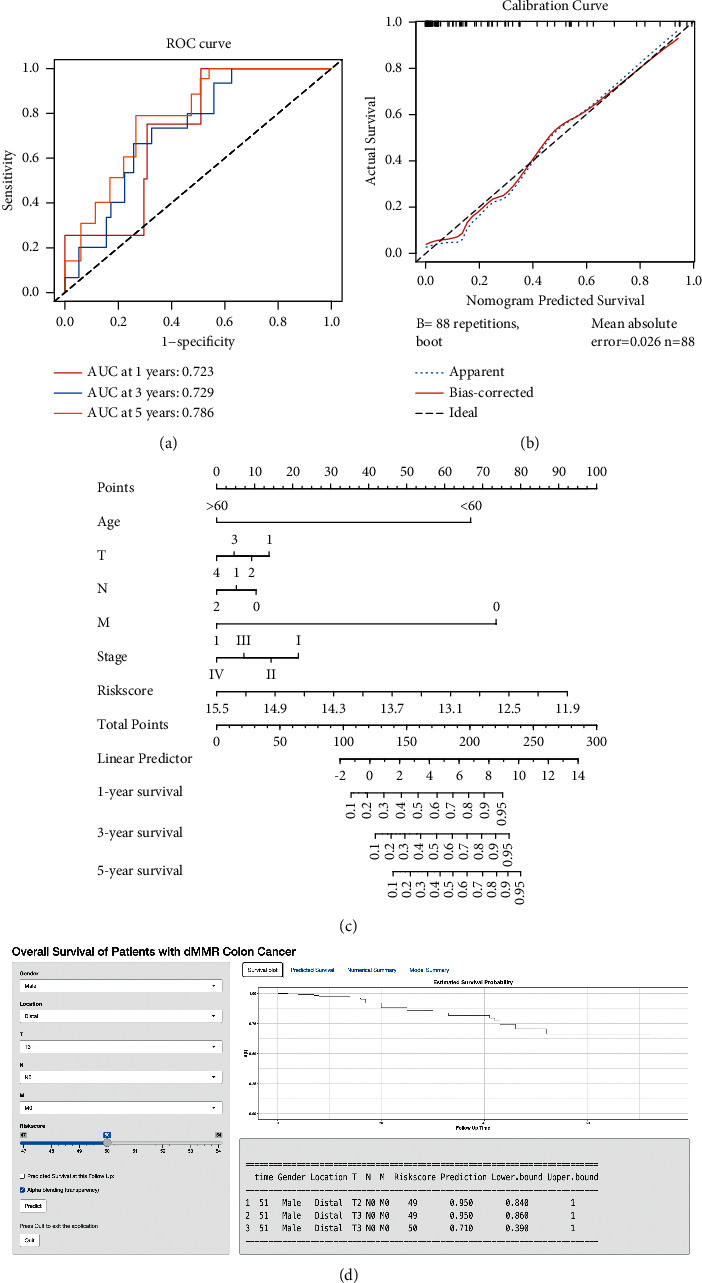
The validation and visualization of the prognostic model. (a) The ROC curve showed that the discrimination of the model was valuable for the prediction of 1-, 3-, and 5-year OS. (b) The calibration plot suggested that the performance of this model was stable. (c) The nomogram was built based on the risk score and the clinicopathological features, including age, pathological *T*, *N*, *M*, and stage. (d) The shiny app was developed based on the prognostic model for convenient clinical application.

We used ROC and the calibration model to evaluate the discrimination ability and stability of this prognostic model. The result of ROC analysis suggested that this model had a good discrimination ability to predict 1- (AUC = 72.3%), 3-(AUC = 72.9%), and 5-year (78.6%) OS for dMMR CC patients (Figure 7(a)). The apparent and bias-corrected curves were close to the ideal curve in the calibration plot, indicating good stability and consistency of this prognostic model (Figure 7(b)).

Subsequently, we developed a nomogram for clinical application. Along with the risk score of the prognostic model, it also contained pathological *T*, *N*, *M*, stage, and age (Figure 7(c)). Its usage was quite simple and user-friendly, which was divided into three steps. First, each factor would be read and would have a different point according to the point scale. Second, the point of each factor would be added up to have a total point. It would be identified on the total point scale. Finally, the OS of dMMR CC patients would be represented in the probability scale, based on the total point calculated in the previous step.

Moreover, to make the diagnostic model more convenient to use, we developed an online dynamic nomogram (Figure 7(d)). It could simplify the four-step usage into a two-step usage. First, different values of each clinicopathological factor could be chosen in the drop-down menu. Then, the user only had to click the predict button, and the OS probability of the patients would be calculated automatically based on the prognostic model. Meanwhile, a forest plot with a 95% confidence interval (CI) would be simultaneously visualized.

## 4. Discussion

In this study, based on hypoxia-related gene signature, we have classified dMMR CC patients into 2 subgroups. Most hypoxia-related genes in HS were increasingly expressed, compared to those in NS. Therefore, we considered that NS was a normoxia subgroup and HS was a hypoxia subgroup. The TIME of NS was quite different from that of HS, showing that the infiltration of most TILs was significantly higher in NS. Meanwhile, it also had lower expressions of the immune checkpoint molecules and chemokines. These results indicated that NS might have a relative normoxia TME, which could promote the infiltration of TILs, thus resulting in a better prognosis. Moreover, we also developed a prognostic model based on two hypoxia-related genes, namely, MT2A and MAFF. It showed a stable and consistent predicting ability, also indicating the close association between hypoxia and prognosis of patients with dMMR CC. Finally, we identified that MAFF might be a potentially important regulatory gene involved in the hypoxia TME of dMMR CC patients.

The MSI/dMMR CC patients were initially considered to have high levels of TMB and neoantigens, which could induce sustained self-immune responses and ensure the curative effect of immunotherapy [8]. At present, immune checkpoint inhibitors (ICIs) have been approved by FDA to treat advanced MSI/dMMR CC patients [38]. However, a previous study has shown that there were still nearly 60% MSI/dMMR that were not responsive to ICIs, indicating that the TIME was variable even among MSI/dMMR patients [39]. Therefore, the urgency is to uncover the underlying mechanism and identify the most responsive patients, thus improving therapeutic efficiency.

In this study, we found that even among patients with CC with dMMR status, there was variable TIME, which was considered to be closely associated with the therapeutic efficiency of ICIs and prognosis. A previous study suggested that alterations and evolutions had always existed inside the TME of the tumors treated with ICIs [40]. Meanwhile, exhaustion and deletion of tumor-specific CD8+ T cells could significantly impair the antitumor effect of ICIs and induce drug resistance [41]. These findings highlight the importance of CD8+ T cells involved in the therapy of ICIs. However, the uncontrolled growth of tumor cells creates hypoxia and malnutritional TME. It will significantly impair the metabolism and colonization of CD8+ T cells, thus inducing tumor progression and drug resistance of ICIs [10]. In this study, patients in the normoxia subgroup had significantly higher infiltration of CD8+ and CD4+ T cells. We supposed that these patients might be more responsive to ICIs, and the better prognosis of them might be partially associated with higher infiltration of TILs. Interestingly, in NS, besides higher infiltration of TILs, there was also significantly lower infiltration of neutrophils. A previous study suggested that neutrophils could act as either a tumor suppressor or a promotor, depending on tumor type and stage [42]. Later in the progression of tumors, neutrophils would promote tumor growth by releasing vascular endothelial growth factor (VEGF) to stimulate angiogenesis [42]. In this study, we also found that the expression of VEGF was significantly higher in HS accompanied by the higher infiltration of neutrophils. In colorectal cancer (CRC), higher infiltration of tumor-infiltrating neutrophils was positively associated with higher histological grade, advanced pathological stage, and poorer recurrence-free survival [34]. Besides, in both GEO sets and TCGA cohort, we have identified higher infiltration of M1 and lower infiltration of M2 in NS. High-mobility group box 1 protein (HMGB1), increasingly expressed in hypoxia TME and closely associated with the development of CC, has been proven to be related to macrophage colonization, especially for the M2 type [43]. These M2 macrophages would secrete high levels of VEGF and TNF-*α*, which was also proven in this study in Figure 3(b), and consequently promote the progression and metastasis of tumors [44, 45].

PD-1, a type of inducible membrane protein, is often upregulated with the activation of CD8+ T cells *via* NFATc1, Notch, and STAT pathways [46–48]. However, tumor-infiltrating CD8+ T cells will experience distinct differential reprograms. Some of them subsequently acquire an exhaustion type, due to the hypoxia, acidic, and malnutritional TME. During the reprogramming process, these cells will elevate the inhibitory receptors, such as PD-1 [49]. These findings highlight the importance of PD-1 induction in CD8+ T cells in the TME and thus open a door to PD-1 inhibitor with therapeutic effect in clinical application. In this study, we found that PDCD1, another symbol of PD-1, was increasingly expressed in NS. Except for PD-1, all the other immune checkpoint molecules, including PD-L1, CTLA4, et al., declined in NS. Previous studies also identified that hypoxia could induce the high expression of immune checkpoint molecules, such as indoleamine 2,3-dioxygenase (IDO) and PD-L1 [50, 51]. The expression of immune checkpoint molecules might be negatively associated with the infiltration of TILs and the prognosis in types of cancers [7, 52, 53].

In hypoxia TME, CXC chemokines are mainly secreted by tumor-associated macrophages (TAMs) and myeloid-derived suppressor cells (MDSCs) *via* hypoxia-inducible factor (HIF)/nuclear factor *κ*B (NF-*κ*B) pathway [54, 55]. Subsequently, these activated CXC chemokines will promote the progression and metastasis of cancer *via* numerous protumor properties. Seven CXC chemokines reported in this study, such as CXCL1, CXCL2, CXCL3, CXCL5, CXCL6, CXCL8, and CXCL16, are all angiogenic [56–59]. High expression of these chemokines will cause neovascularization, thus promoting tumor invasion and metastasis [60]. In this study, we also found that the hypoxia subgroup had a higher expression of CXC chemokines, which might be related to the poorer prognosis of this subgroup.

In this study, three genes, namely, MT1E, MT2A, and MAFF, were the intersection of GEO-DEGs, TCGA-DEGs, and hypoxia-related gene list. Two of them, namely, MT2A and MAFF, were included in the construction of the prognostic model for clinical application. Metallothioneins (MTs), a family of low molecular weight proteins, play an important role in the regulation of the cellular homeostasis of zinc and copper [61]. Among types of MTs, MT1 and MT2 are the most widely distributed isoforms, which were commonly found in many tissues, especially in the liver and kidneys [62]. MTs may be tightly involved in carcinogenesis, including tumor growth, differentiation, angiogenesis, and metastasis [63]. Meanwhile, MTs can also be involved in the process of TME remodeling and immune escape by binding to the plasma membrane of TILs and changing their immunomodulatory functions [64]. High levels of MTs released in the extracellular environment were proven to be closely associated with immunosuppression, tumor aggressiveness, and metastasis in numerous cancers [65–67]. Another hub gene, MAFF, is also hypoxia related and has been proven to be tightly associated with invasion and metastasis of cancer *via* HIF/NF-*κ*B pathway [68]. Meanwhile, it has also been identified to promote M2 polarization in TAMs, indicating its role involved in TME remodeling and ICI therapy [69]. In this study, we also found that higher expression of MT1E, MT2A, and MAFF was related to the poorer infiltration of CD8+ T cells and poorer prognosis of dMMR CC patients. In a further study, we decided to focus on these genes and their roles involved in the development and progression of CC.

Moreover, we also developed a prognostic model, as well as a nomogram and a shiny app, based on the hypoxia-related genes. Numerous studies have also constructed similar prognostic models in types of cancer, such as osteosarcoma [70], breast [71], renal [72], and lung cancer [73]. In all these studies, despite different genes, patients with higher expression of hypoxia-related genes had either poorer pathological stage, infiltration of immune cells, or survival status, highlighting the potential of hypoxia genes in clinical application.

This study has several limitations: (1) due to limiting data resources of patients with CC with dMMR, the sample size of this study was small, which might induce selection bias; (2) during the validation process of the prognostic model, external validation set was not available, thus inducing risks of instability of the model; (3) it is only a bioinformatic study without experiment in vitro/vivo; however, we have identified three hypoxia-related hub genes that played an important role in TIME and prognosis of dMMR CC patients. In a further study, we will focus on these genes and their biological functions in the development and progression of dMMR CC.

In summary, we have classified dMMR CC patients into hypoxia and normoxia subgroups and revealed their different TIMEs. We found that the hypoxia subgroup had lower infiltration of TILs, more expression of immune checkpoint molecules and chemokines, and a poorer prognosis. Based on all the evidence, we supposed that hypoxia TME might be potentially associated with ICI resistance in dMMR CC patients. Subsequently, we identified two hub genes and will focus on them in further study. Moreover, we have developed a prognostic model. Based on it, a nomogram and a shiny app were constructed for clinical application.

## Data Availability

The generated and analyzed databases of the present study are freely available in GEO (https://www.ncbi.nlm.nih.gov/geo/) and TCGA (https://portal.gdc.cancer.gov/) databases.

## Supplementary Materials

Supplementary Materials Supplementary 1. Table S1: the list of 200 hypoxia-related genes. Supplementary 2. Table S2: the list of 22 hypoxia-related genes with prognostic value. Supplementary 3. Table S3: different clinicopathological features between normoxia and hypoxia subgroups in GEO and TCGA sets. Supplementary 4. Figure S1: the validation of the hypoxia-related gene clustering method in the TCGA COAD cohort. (a) The heatmap showed different expressions of 22 prognostic hypoxia genes between the two subgroups. (b) There were 98 DEGs between two subgroups. Among them, 6 genes were hypoxia related. (c) The result of CIBERSORTx analysis showed different infiltration of immune cells between two subgroups. (d) The heatmap showed different expressions of immune-related genes between two subgroups. Despite PDCD1, all the other immune checkpoint molecules (e) and chemokines (f) were increasingly expressed in HS.
