# Supplementary material for: Distinct Hypoxia-Related Gene Profiling Characterizes Clinicopathological Features and Immune Status of Mismatch Repair-Deficient Colon Cancer
Source: J Oncol. 2021 Dec 7;2021:2427427. doi: 10.1155/2021/2427427 (PMC8670907; doi:10.1155/2021/2427427)
Supplement: Supplementary Materials — Supplementary 1. Table S1: the list of 200 hypoxia-related genes. Supplementary 2. Table S2: the list of 22 hypoxia-related genes with prognostic value. Supplementary 3. Table S3: different clinicopathological features between normoxia and hypoxia subgroups in GEO and TCGA sets. Supplementary 4. Figure S1: the validation of the hypoxia-related gene clustering method in the TCGA COAD cohort. (a) The heatmap showed different expressions of 22 prognostic hypoxia genes between the two subgroups. (b) There were 98 DEGs between two subgroups. Among them, 6 genes were hypoxia related. (c) The result of CIBERSORTx analysis showed different infiltration of immune cells between two subgroups. (d) The heatmap showed different expressions of immune-related genes between two subgroups. Despite PDCD1, all the other immune checkpoint molecules (e) and chemokines (f) were increasingly expressed in HS. [file 2427427.f1.zip › 2427427.f1/Table S3.docx]

| Table S3. Different clinicopathological features between normoxia and hypoxia subgroups in GEO and TCGA sets. | | | | | | | | | | |
| --- | --- | --- | --- | --- | --- | --- | --- | --- | --- | --- |
| **Characteristics** | **GEO** | | | | | **TCGA** | | | | |
|  | **Total** | **NS (12)** | **HS (76)** | **t/X^2^/Z** | ***p*** | **Total** | **NS (36)** | **HS (13)** | **t/X^2^/Z** | ***p*** |
| Gender |  |  |  | 0.288 | 0.591 |  |  |  | 1.387 | 0.175 |
| Female | 45 | 7 | 38 |  |  | 30 | 20 | 10 |  |  |
| Male | 43 | 5 | 38 |  |  | 19 | 16 | 3 |  |  |
| Age |  | 43.72 ± 15.57 | 71.50 ±1.64 | -6.169 | 0.000 |  | 55.14±12.40 | 60.23±15.12 | -1.197 | 0.237 |
| Tumor location |  |  |  | 0.890 | 0.345 |  |  |  |  |  |
| Proximal | 68 | 8 | 60 |  |  |  |  |  |  |  |
| Distal | 20 | 4 | 16 |  |  |  |  |  |  |  |
| Stage* |  |  |  | -2.075 | 0.038 |  |  |  | -3.961 | 0.030 |
| I | 13 | 6 | 7 |  |  | 9 | 9 | 0 |  |  |
| II | 43 | 3 | 40 |  |  | 17 | 12 | 5 |  |  |
| III | 30 | 2 | 28 |  |  | 15 | 12 | 3 |  |  |
| IV | 2 | 1 | 1 |  |  | 8 | 3 | 5 |  |  |
| T* |  |  |  | -1.087 | 0.277 |  |  |  | -4.668 | 0.198 |
| T1 | 3 | 2 | 1 |  |  | 1 | 1 | 0 |  |  |
| T2 | 12 | 4 | 8 |  |  | 9 | 9 | 0 |  |  |
| T3 | 45 | 1 | 44 |  |  | 34 | 23 | 11 |  |  |
| T4 | 28 | 5 | 23 |  |  | 5 | 3 | 2 |  |  |
| N* |  |  |  | -0.491 | 0.624 |  |  |  | -2.074 | 0.355 |
| N0 | 56 | 9 | 47 |  |  | 26 | 18 | 8 |  |  |
| N1 | 17 | 0 | 17 |  |  | 15 | 13 | 2 |  |  |
| N2 | 15 | 3 | 12 |  |  | 8 | 5 | 3 |  |  |
| M* |  |  |  | 2.298 | 0.130 |  |  |  | 6.346 | 0.012 |
| M0 | 86 | 11 | 75 |  |  | 41 | 33 | 8 |  |  |
| M1 | 2 | 1 | 1 |  |  | 8 | 3 | 5 |  |  |

Note: * Ranked data were analyzed using the Mann-Whitney U test.
